# On the Traceability of Commercial Saffron Samples Using ^1^H-NMR and FT-IR Metabolomics

**DOI:** 10.3390/molecules21030286

**Published:** 2016-02-29

**Authors:** Roberto Consonni, Stella A. Ordoudi, Laura R. Cagliani, Maria Tsiangali, Maria Z. Tsimidou

**Affiliations:** 1 Institute for Macromolecular Studies, NMR Laboratory, National Research Council, v. Corti 12, 20133 Milan, Italy; lauraruth.cagliani@ismac.cnr.it; 2 Laboratory of Food Chemistry and Technology (LFCT), School of Chemistry, Aristotle University of Thessaloniki (AUTh), 54124 Thessaloniki, Greece; steord@chem.auth.gr (S.A.O.); mariatsiangali@outlook.com.gr (M.T.); tsimidou@chem.auth.gr (M.Z.T.)

**Keywords:** saffron traceability, saffron mislabeling, FT-IR, NMR, metabolomics

## Abstract

In previous works on authentic samples of saffron of known history (harvest and processing year, storage conditions, and length of time) some biomarkers were proposed using both FT-IR and NMR metabolomics regarding the shelf life of the product. This work addresses the difficulties to trace back the “age” of commercial saffron samples of unknown history, sets a limit value above which these products can be considered substandard, and offers a useful tool to combat saffron mislabeling and fraud with low-quality saffron material. Investigations of authentic and commercial saffron samples of different origin and harvest year, which had been stored under controlled conditions for different lengths of time, allowed a clear-cut clustering of samples in two groups according to the storage period irrespectively of the provenience. In this respect, the four-year cut off point proposed in our previous work assisted to trace back the “age” of unknown samples and to check for possible mislabeling practices.

## 1. Introduction

Saffron, the spice that is exclusively produced from the stigmas of the *Crocus sativus* L. flower after drying under different conditions [1] is the most expensive spice in the world and, consequently, the target of frequent fraud and mislabeling practices [2]. Mislabeling is a serious fraudulent practice reported for a series of precious food products and it is related to fake information provided along the trade chain. Fake information on packaged saffron can be related to the genuineness of its content, origin, Protected Designation of Origin (PDO) name, as well to the “best before” date or harvest year that often appears on the packaging. To this view, new scientific criteria are sought for examining authenticity, origin, and traceability aspects of this spice [3] as its price per kilo is the highest among all food items and not only among spices.

During the last years, the chemical analysis of mixtures, such as food extracts, moved from the classical approach based on chemical isolation followed by structure elucidation toward metabolomics. This switch allows the identification and quantification of all molecules present in a biological system, representing a powerful tool in food science for monitoring, in a wider context, product quality and authenticity [4,5,6]. In this respect high-resolution nuclear magnetic resonance (HR–NMR) spectroscopy represents a unique and versatile technique to investigate foods, providing detailed information at molecular level. As a matter of fact, a single experiment provides the identification of a large number of different classes of chemical compounds leading to both qualitative and quantitative analysis. Moreover NMR is a non-invasive, highly reproducible, and fast technique. Even if the sensitivity of the conventional NMR spectrometers could be as low as 1 μM a good estimate of the main metabolites content can be achieved obtaining an almost full picture of the matrix analyzed [7]. On the other hand, FT-IR spectroscopy has a strong potential in the analysis and quality control of foods because of its sensitivity, versatility, and rapidity [8]. Depending on the measurement method used (e.g., transmission, diffuse reflectance, attenuated total reflectance) FT-IR spectroscopy minimizes, or even eliminates, sample preparation. Indeed, applications of mid-infrared (MIR) spectroscopy to food safety, end-product quality/process control, and authenticity studies have drastically increased during the last decade [9]. Although not quite helpful for structure elucidation, infrared bands in the mid region (MIR, 4000–400 cm^−1^) are more informative than those in the near-infrared one (NIR, 10,000–4000 cm^−1^) where broad overtone and combination absorption bands occur [8,10]. In addition, absorptions in the region between 1500–400 cm^−1^, due to bending and skeletal vibrations, are sample-specific and are used as a fingerprint [9]. So far, applications of FT-MIR spectroscopy in the analysis of herbs and spices are still extremely limited [11].

Both NMR and FT-IR analysis result in a huge amount of data that require the use of multivariate statistical analysis to reduce dimensionality and to evaluate variability in samples, leading to sample classification or predictive models.

NMR spectroscopy has been initially applied to characterize individual constituents isolated from saffron extracts for identification purposes [12,13,14,15]. In late 2009, Yilmaz *et al.* [16] published the first NMR work based on the metabolic fingerprinting of methanolic saffron extracts with the aim of distinguishing authentic Iranian from commercial saffron samples obtained from retail stores in Denmark, Sweden, and Turkey. Successively, Cagliani *et al.* [17] investigated the NMR metabolic profiles of dimethylsulfoxide saffron extracts of Italian PDO samples and products available in the Italian market, leading to a clear sample discrimination and highlighting picrocrocin and crocetin esters as the most relevant compounds for the characterization of Italian PDO saffron, whereas fatty acids were found to be the prominent constituents in the commercial samples.

Saffron authenticity and quality issues have been only recently assessed using FT-IR-based metabolomics approaches. More specifically, in 2009 Anastasaki *et al.* [18] were the first to report an application of MIR to traded saffron using the micro-DRIFTS method of measurement along with chemometrics (PCA). Their study aimed to differentiate authentic saffron samples originated from four countries (Iran, Spain, Italy, Greece); an objective that had been investigated using FT-NIR some years ago [19] with the ultimate goal to provide tools for identification of mislabeling of geographical origin. More recently, Ordoudi *et al.* [20] extended the application of the FT-MIR coupled with chemometrics (PCA) to the quality control of traded saffron and, more specifically, for the assessment of saffron “freshness”. The authors suggested that specific infrared bands in the sugar region (e.g., 1028 and 1175–1157 cm^−1^) associated with the presence of glucose moieties and breakage of glycosidic bonds, are useful tools to monitor storage effects on saffron quality and, consequently, that was the first attempt to trace back the “age” of saffron using FT-IR metabolomics. Further insight to the nature and fate of the glycosidic bonds during storage was then gained using a ^1^H-NMR-based metabolomics approach [21]. In that study, ^1^H-NMR data of 98 saffron samples of different origin and harvest year, stored under different conditions for various periods of time, were subjected to OPLS-DA. The chemometric analysis showed that crocetin esters and picrocrocin, being abundant in “fresh” samples, can be considered as biomarkers for saffron stored for less than four years after processing. These results strengthened the opinion that evolution of hydrolytic degradation of crocetin esters and picrocrocin represents a decisive factor for saffron quality deterioration, as already observed by using FT-IR [20]. On the other hand, NMR findings pointed out that α-glucose and α-gentiobiose in unbound form characterized the “non-fresh” samples. Fatty acids, not usually examined in quality control studies of saffron, were also shown to be critical markers for the identification of the latter ones. Hence, a clear-cut clustering of samples in two groups according to their storage history was evidenced and the obtained validated statistical model was able to predict whether a saffron sample can no longer be considered as “fresh”. 

The present study aims at tracing back the “age” of various saffron samples (*n* =17) purchased from open markets and retail shops in major saffron-consuming countries by taking advantage of the so far results from NMR and FT-IR-based metabolomics studies. The corresponding spectra were subjected to chemometric analyses (PCA, PLS-DA, OPLS-DA) in order to classify the samples according to their age. Where possible, the results were interpreted with regard to packaging and “expiration” or “best before” dates on product labels. Classification of samples according to the ISO 3632 quality categories [22] or according to their actual content in major metabolites (crocetin esters and picrocrocin) was also carried out using conventional techniques (UV–VIS, HPLC–DAD). This is the first report on the traceability of commercial saffron samples performed by a combined approach of FT-IR and ^1^H-NMR metabolomics. Our approach is expected to be considered in a forthcoming revision of the ISO 3632 technical standard.

## 2. Results and Discussion

### 2.1. UV–VIS and HPLC–DAD Analysis

The 17 commercial samples labeled as “saffron” were first examined for their purity on the basis of HPLC–DAD analysis, as described under Experimental Section. The typical chromatographic profiles of authentic saffron at 440, 310, and 250 nm were produced in all cases, showing that the commercial samples contained five to six crocetin esters (*trans* or/and *cis*- form) and picrocrocin along with some flavonoid glucosides at varying levels. Constituents of unknown origin were not detected under the conditions of this analysis in any of the examined samples. In detail, it was found that the total content in crocetin esters varied between 9.1 to 27.1 g/100 g of saffron (expressed as *trans*-4-digentiobiosyl ester of crocetin or *trans*-4-GG) while the main ester, namely *trans*-4-GG, oscillated between 4.8 and 14.3 g/100 g of saffron. The samples were also found to contain picrocrocin at levels between 7.1–14.2 g/100 g of saffron. Taking into account literature data, the latter values correspond to samples of relatively high coloring strength [23,24]. Classification of the samples according to ISO 3632 trade specifications showed that although the criterion of minimum picrocrocin content value for classification to commercial category I (premium quality) (E^1%^_257nm_ > 70) was fulfilled in all cases, half of the samples belonged to categories II and III on the basis of coloring strength values. These results are shown in Table 1.

Practically, classification to premium trade quality (E^1%^_440nm_ > 200) was made for those samples with an actual content of total crocetin esters higher than 14% *w*/*w*. Previously reported data about correlation of coloring strength values with the approximate content in crocetin esters seem to support our observation [25]. These results show that a considerable number of the commercial samples analyzed soon after their purchase were of inferior commercial quality but not substandard. All of these samples were then stored at room temperature, in the dark and then re-examined using FT-IR and ^1^H-NMR techniques.

### 2.2. FT-IR Analysis

**Figure 1:**
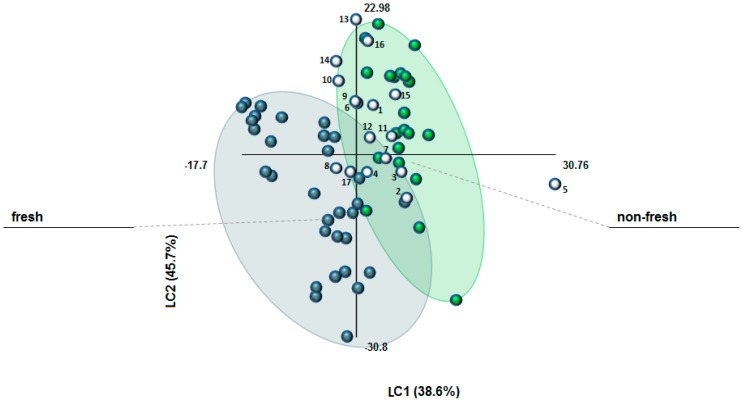
Scatterplot of PLS–DA model performed considering 52 authentic saffron samples of different geographical origins, harvest year, and stored under different conditions for different periods (“fresh”: 0–4 years of storage and “non-fresh”: 7–12 years of storage). LC1 = 38.6%, LC2 = 45.7%, R^2^ (Y) = 78%, Q^2^ = 77%. Blue, green, and white dots represent “fresh”, “non-fresh”, and the 17 commercial samples of unknown storage history, respectively.

The FT-IR database of authentic “fresh” (stored for less than four years after processing) and “non-fresh” (stored for 7–12 years) samples (see Supplementary Material, Figures S1–S4) was used to build a PLS-DA model, properly validated, which has been successively exploited for the classification of 17 commercial samples of unknown history. The classification criteria were based on t1 and t2 cut-off values of t1 > 2.0 and t2 > 6.0 for “non-fresh” saffron as well as t1 ≤ 0 and t2 ≤ −1 for “fresh saffron” (see Supplementary Material, Figure S4b). Figure 1 illustrates the results of this study (April 2015). As it can be observed, only two out of the 17 samples (n° 8 and 17) were allocated to the group of “fresh” saffron. On the other hand, two samples (n° 1 and 15) were clearly characterized “non-fresh” and other four ones (n° 5, 7, 11, and 12) fulfilled only the criterion of t1 ≥ 2.0 for “non-fresh” saffron. Four samples (n° 10, 13, 14, and 16) presented exceptionally high t2 values (> 12.0) so that they were rather allocated to “non-fresh” saffron, as well. The rest of the samples (n° 2–4, 6, and 9) could not be clearly classified on the basis of t1 and t2 values. Our findings traced that most of the commercial saffron samples were actually very close to or already had passed the cut-off point of four years after harvest and processing (see also Supplementary Material, Table S1). This would mean that, with few exceptions, the commercial samples were already unfit for delivering their highly desirable sensorial and functional properties upon human consumption [26].

### 2.3. NMR Analysis

The ^1^H-NMR spectra of the 17 commercial samples were recorded (June 2015) and the data were re-projected in the OPLS-DA model developed and validated in a previous work [21] to evaluate their freshness. The model originally constituted by 98 samples representing two classes of samples according to the storage period of 0–4 years (“fresh”) and of 5–14 years (“non-fresh”). Each of the 17 commercial samples was classified by a classification score (Y-predicted), indicative of its representativeness. In our case a threshold value of 0.6 has been chosen as a good classification score; the resulting classification list is reported in Table 2. In details, five out of the 17 samples (n° 3, 6, 8, 9, and 17) were classified as “fresh” (stored under optimum conditions for less than four years after harvest) while six out of the 17 samples (n° 5, 11, 12, 13, 15, and 16) were clearly indicated as “non-fresh”. Samples 1, 2, 4, 7, 10, and 14 were most likely of intermediate state of storage and quality (corresponding to 4–5 years of storage under optimum conditions).

The results of this prediction were in partial agreement with the FT-IR-based findings. As a matter of fact, samples 8 and 17, as well as samples 5, 11–13, 15, and 16, were clearly described as “fresh” and “non-fresh” respectively, using both metabolomics approaches; it was also confirmed that more than four years had passed after harvest and processing of samples 2 and 4. However, there were cases of samples for which the two approaches led to different results concerning their freshness. All of these findings were then combined and critically evaluated according to the label of the packed products wherever available. 

In details, sample 8, purchased from an open market in Iran, had no information about its production or expiration date. On the other hand, samples 3 and 17, as well as sample 6, which had been classified as “fresh” based on NMR-data, were all bought in Qatar; the former two claimed Iranian origin while the latter was labeled as of Spanish origin. These three samples also claimed a two-year period of usage after manufacturing. According to their “best before” date sample 17 had already expired while the other two ones would be expected to expire in approximately six months after the date of NMR analysis, but our traceability findings suggested that these products were most probably of premium quality at the time of packing and could last even longer than the declared date. Sample 9 was bought in an Italian market in powder form. Only the “best before” date was labeled claiming that the product would expire almost 1.5 years after the NMR analysis. Our results traced that this information was not misleading to consumers.

Concerning the samples characterized as “non-fresh” using both FT-IR and NMR-based metabolomics approaches, the available labeling information was quite enlightening. In particular, sample 11—bought in an Israeli market and claiming Spanish origin and crop year of 2011 (four years before analysis)—claimed to be “best before” the end of 2017, that is, more than two years after FT-IR and NMR analyses. Our findings traced that this information was rather misleading. Additionally, the particular sample had already presented inferior trade quality since a short period after its purchase (see Table 1). The same suggestion can be made for the labeling of sample 13 which was in Arabic, claimed Iranian origin and recommended a three-year period of usage after packing date (end of 2012). Samples 15 and 16 were bought in Qatar. The former did not contain any indication about the origin, packing, or expiration date, while the latter claimed Iranian origin and a two-year period of storage, although exact packing and “best before” dates were unclearly stated. Sample 12 that was bought in an open Israeli market with no labeling information about origin, packing, or expiration date was also classified as “non-fresh”. It has to be pointed out that sample 5, of Iranian origin and an indication for two-year storage period after its production (early 2013), was the only classified “non-fresh” sample that had already expired before spectroscopic analyses, according to the product label information.

Six out of the seventeen studied commercial samples could not be clearly classified in the two groups on the basis of NMR analysis, as mentioned above. In particular, samples 1 and 2 that claimed as packing dates the ninth and tenth months of 2015, respectively, and a two-year storage period, were not found properly classified to either of the two sample categories. This could imply that their processing date was most probably traced back to 2011, or even earlier. Furthermore, sample 10, labeled as Italian and in powder form that could be considered fresh on the basis of its “best before” date (2016), was not found to belong to the ”fresh” group by any of the two metabolomics approaches. The fact that it had been packed in powder form, which facilitates quality deterioration, could also account for this finding. Another point of importance was indicated by the study of sample 4. This sample was of the same production company and manufacture date as sample 5 (identified as “non-fresh”) but not clearly classified by either FT-IR or NMR analyses. The same sample had presented much higher coloring strength value than that of sample 5 soon after their purchase (see Table 1). This last finding can be attributed to different quality of the sample upon packing or even different storage conditions until purchase. Sample 7, of unknown origin and labeled to be packed in Australia, was already expired. We assume that this latter information is truthful since the sample was not found “fresh” by either of the two metabolomics approaches of this study. Sample 14 bought in Qatar and claiming Iranian origin did not bear any labeling with regard to its production or “best before” dates and was also found “non-fresh” by both FT-IR and NMR analysis.

After the acquisition of FT-IR and NMR data and evaluation of the results on the basis of labeling information, HPLC-DAD analysis of the commercial samples was performed again to measure the remaining contents in total crocetin esters, *trans*-4-GG and picrocrocin and to find some possible correlation with traceability findings. Samples 9 and 10 were not reanalyzed, as there was no leftover amount. Our results showed that samples 3, 6, 8, and 17 (clearly classified as “fresh” according to NMR findings) along with sample 4 (not clearly classified by both FT-IR and NMR analysis) contained more than 14.0% *w*/*w* of crocetin esters. This observation is in line with what was suggested for the samples of high coloring strength and, thus, premium trade quality in the beginning of this study. For these five samples, the actual content of the major crocetin ester (*trans*-4-GG) was found higher than 8.0% *w*/*w*, while picrocrocin levels ranged from 11.0% to 14.2% *w*/*w*. Noticeably, samples 1 and 2, initially rich in total crocetin esters and picrocrocin, did not retain their high quality during storage in the laboratory.

Classification of the commercial samples into the ISO 3632 quality categories ([22] was finally carried out as a means to interpret the information derived from metabolomics studies in terms of current trade specifications. The results are presented in Table 3.

From these data, it can be suggested that those samples that had been characterized as “non-fresh” by both FT-IR and NMR metabolomics presented much lower E^1%^_257nm_ and E^1%^_440nm_ values (related with the picrocrocin and total crocetin ester contents, respectively) than the minimum limits for classification to quality category I. Actually, only few of these samples were classified to the lowest quality category (III) while the rest were substandard. On the other hand, all of the samples that were still characterized “fresh” based on NMR data, seemed to retain moderate coloring strength values that would classify them to the quality categories I and II. Again, no useful information could be derived from the E^1%^_330 nm_ values. 

Overall, it seems that the spectroscopic databases developed within the SaffronOMICS COST Action FA1101 frame represent an affordable and rapid tool that can be used to impartially evaluate the information reported on the label of the traded products as saffron. In this view, traceability requirements in the international transactions of saffron can be checked at various control points. A first screening of sample freshness could be made using FT-IR but, in cases of uncertainty, examination by ^1^H-NMR is expected to provide more conclusive information. There is an obvious need for frequent updating of the databases as required for other foodstuffs. The date of packaging, in the best of our expectations, should refer to production of the latest harvest or of the harvest of the year before that. Considering that most of the commercial samples report the expiring date set two years after the packaging date, it seems that in practice four years after harvesting/processing date is a critical length of period for saffron trade. 

## 3. Experimental Section

### 3.1. Saffron Samples

Commercial saffron samples (*n* = 17) of unknown storage history have been analyzed for the evaluation of their age after processing. After their delivery to LFCT, the samples, in packings or in bulk, were stored at room temperature in the dark. All the information concerning these samples is reported in Table 4. 

### 3.2. Standards, Reagents, and Solvents

*Trans*-4-GG crocetin ester was laboratory-isolated by semi-preparative reversed phase-high performance liquid chromatography (RP-HPLC) as previously described in detail by Kyriakoudi and Tsimidou, 2015 [27]. Purity of isolated *trans*-4-GG (97%) was checked (a) chromatographically by RP–HPLC–DAD in the range of 200–550 nm and calculated as the percentage of the total peak area at 440 and (b) by nuclear magnetic resonance (NMR) spectroscopy recording the 1D ^1^H spectra at 300 MHz on a Bruker 300AM spectrometer (Rheinstetten, Germany).

Acetonitrile, methanol (Chem-Lab, Zedelgen, Belgium) and acetic acid (Fluka Chemie, Buchs, Switzerland) used were of HPLC grade. Ultrahigh purity water was produced using a SG Ultra Clear Basic UV system (SG Wasseraufbereitung und Regenerier station GmbH, Barsbüttel, Germany). Deuterated dimethylsulfoxide (DMSO-*d*_6_, 99.96 atom% D) was purchased from Euriso-Top (Saclay, France).

### 3.3. Extraction of Crocetin Esters and Picrocrocin

Methanol-water extracts of ground stigmas or powder were prepared according to the ISO/TS 3632–2 method [28] with slight modifications. Briefly, 0.1 g was mixed with methanol–water (1:1, *v*/*v*) in a 200 mL volumetric flask. Crocetin esters and picrocrocin were extracted by rigorous agitation (1000 rpm) for one hour at ambient temperature (25 °C) away from direct sunlight. Prior to analysis, an aliquot from the extract was diluted (1:10) with methanol–water (1:1, *v*/*v*) and the corresponding solutions were filtered through RC-55 filter (13 mm i.d., 0.45 μm pore size).

### 3.4. UV–VIS Spectrophotometric Examination

The UV–VIS spectra of all of the extracts were recorded in the region 200–600 nm with a spectrophotometer (Shimadzu UV 1601, Kyoto, Japan) equipped with quartz cells (1 cm × 1 cm × 4 cm). The results were expressed as E^1%^_λmax_ according to the equation E^1%^_λmax_ =(D × 100)/(m(100 − H)), with D as the absorbance value, m as the mass of the test portion (g), H as the moisture and volatile content of the sample (%, *w*/*w*), λ_max_ for crocetin esters 440 nm, λ_max_ for picrocrocin 257 nm, and λ_max_ for safranal 330 nm, as proposed in the ISO 3632-2 trade standard [28].

### 3.5. Liquid Chromatographic Analysis

High-performance liquid chromatography (HPLC) was used to separate and quantify the crocetin sugar esters and picrocrocin. The HPLC system consisted of a pump, model P4000 (Thermo Separation Products, San Jose, CA, USA), a Midas auto sampler (Spark, Emmen, The Netherlands) and a UV 6000 LP diode array detector (DAD) (Thermo Separation Products, San Jose, CA, USA). Separation was carried out on a LiChroCART Superspher 100 C18 (125 mm × 4 mm i.d., 4 μm) column (Merck, Darmstadt, Germany). The elution system used consisted of a mixture of water/ acetic acid (1%, *v*/*v*) (A) and acetonitrile (B). The linear gradient was 20% to 100% B in 20 min. The flow rate was 0.5 mL/min. The injection volume was 20 μL. The analytical samples were filtrated through a 0.45 μm membrane filter prior to injection. Chromatographic data were processed using the ChromQuest Version 3.0 software (Thermo Separation Products, San Jose, CA, USA). Monitoring was in the range of 200–550 nm and quantification was carried out by integration of the peak areas at 250 nm (picrocrocin) and 440 nm (crocetin esters).

Identification of *trans*-4-GG crocetin ester and picrocrocin was achieved by comparison of retention times, UV−VIS spectra matching with those of available standards and literature data [29,30]. Quantification of crocetin esters and picrocrocin (%, *w*/*w* on dry weight) was based on construction of calibration curves of properly diluted aqueous methanolic solutions: (i) *trans*-4-GG crocetin ester (y = 34891x −774339, 9.5–480 ng/10 μL, R^2^ = 0.99 (*n* = 6), (ii) picrocrocin (y = 9524.8x + 416788, 90–913.5 ng/10 μL, R^2^ = 0.99) (*n* = 5).

### 3.6. FT-IR Analysis

#### 3.6.1. Sample Preparation

All samples were mixed with KBr at a 1/180 ratio (*w*/*w*) and homogenized. This mixture (0.181 g) was then compressed under a pressure of ca. 200 MPa for 1 min to form a thin KBr disc. For each sample, the disc preparation procedure was carried out in triplicate. 

#### 3.6.2. Data Acquisition and Pre-Processing

FT-IR spectra were obtained using a Shimadzu IRAffinity-1 (Shimadzu Europa GmbH, Duisburg, Germany) spectrometer operating in the region 4000–400 cm^−1^ in the absorbance mode. A total of 64 scans with 4 cm^−1^ resolution were acquired for each spectrum. The spectrum of a clean KBr disc (without sample) was used for background subtraction. The spectrometer was located in an air-conditioned room (25° C). The spectra were stored using the software IRsolution (version 1.50) supplied from the same manufacturer. All spectra were smoothed by 15 points using the software function “smoothing action”. Then the baseline was corrected using the “multipoint baseline operation” facility (set zero at 400, 870, 1880, and 4000 cm^−1^). Finally, the spectra were normalized using the order “normalize action” so that the minimum absorbance was set at Abs = 0 (zero) and the maximum at Abs = 1. The data obtained were processed further using the tools in the Microsoft Excel 2010 software. Each spectrum was truncated to 1868 data points. From this dataset, intensity values in the regions of 820–1300 and 1600–1800 cm^−1^ (354 data points) were selected for statistical treatment.

#### 3.6.3. Multivariate Statistical Analysis

The add-in Multibase in Excel (Multibase 2015, Microsoft Excel, www.numericaldynamics.com) was used to perform Principal Component Analysis (PCA) and Projection to Latent Structures-Discriminant Analysis (PLS-DA) of the FT-IR spectral data. Data preparation was made using the scaling method of standard deviations. 

Principal Component Analysis (PCA) attempts to simplify the distributions of samples and identify the underlying factors that explain the pattern of variable and sample correlations. Understanding of similarity or dissimilarity of samples using extracted factors is much easier than using unprocessed data. When samples are assigned into some categories in advance, the distributions of the categories is shown with ellipses. Comparing the sample scatterplot with the loadings one, the significant variables which contribute to sample distribution can be easily identified. PLS-DA was performed in order to maximize the separation of categories. It is based on a classical PLS regression, where the response variables are replaced by the set of dummy variables. The dummy variables are defined in such a way that their weights are one and independent (orthogonal) each other. The accuracy of model was evaluated by 200 permutation validations to safely overcome randomness or overfitting to the model.

The distance to the model (DModX) test was applied to check for outliers and evaluate whether the test set samples fall within the model applicability domain. 

### 3.7. ^1^H-NMR Analysis

#### 3.7.1. Sample Preparation

4 mg of sample was extracted with deuterated dimethylsulfoxide (DMSO-*d*_6_, 600 μL), stirred for 3 min at room temperature and, after 10 min, the mixture was centrifuged at 12,100 *g* for 10 min. 500 μL of the supernatant was used for the NMR analysis.

#### 3.7.2. ^1^H-NMR Data Acquisition 

All ^1^H-NMR spectra have been recorded on a Bruker DMX 500 spectrometer (Bruker Biospin GmbH Rheinstetten, Karlsruhe, Germany) operating at 11.7 T and equipped with a 5-mm reverse probe with z-gradient. Spectra were recorded at 300 K, with a spectral width of 8012 Hz and 32 K data points. Residual water suppression was achieved by applying a presaturation scheme with low power radio frequency irradiation for 1.2 s. 

#### 3.7.3. ^1^H-NMR Data Processing

A resolution enhancement function was applied with an exponential function with LB = 0.5 before Fourier transformation. The phase and the baseline were carefully adjusted with TOPSPIN software (Bruker Biospin, GmbH, v 3.0, Rheinstetten, Karlsruhe, Germany). Spectra were aligned with respect to the DMSO residual signal at 2.5 ppm and successively the region between 0.4–10.4 ppm was reduced to buckets, according to the resonance assignment, and integrated. The integral of each bucket was normalized with respect to the total integral area. A residual DMSO signal between 2.48 and 2.53 ppm and residual water signal between 3.30 and 3.37 ppm were set to zero value. (ACD/Spec Manager, ACD Labs, version 11, Toronto, ON, Canada). 

#### 3.7.4. Multivariate Statistical Analysis

The ^1^H-NMR data of the 17 commercial samples under investigation were re-projected in the OPLS-DA (Orthogonal Projection to Latent Structures-Discriminant Analysis) model validated on the corresponding PLS-DA (Projection to Latent Structures-Discriminant Analysis) performed with SIMCA-P+ 13 (Umetrics, Umea, Sweden). For other details see the reference [21].

## 4. Conclusions

In previous works on authentic samples of saffron of known history (harvest and processing year, storage conditions, and length of time) some biomarkers were proposed using both FT-IR and NMR metabolomics regarding the shelf life of the product. In this communication this approach was implemented to trace back the “age” of commercial saffron samples of unknown history. The outcomes were very encouraging as it was possible to set a limit value above which any commercial saffron can be considered substandard. The combination of metabolomic techniques offers a useful tool to combat saffron mislabeling and fraud with low-quality saffron material irrespective of provenience. Overall, the four-year cut off point proposed in our previous work assisted to trace back the “age” of unknown samples and to check for possible mislabeling practices.

## Figures and Tables

**Table 1:** Classification of the 17 commercial samples according to the ISO 3632 quality categories [22] soon after purchase.

Quality Parameters	Samples per Commercial Category		
	I	II	III
	1, 2, 3, 4, 6, 8, 9, 10, 17	7, 14, 16	5, 11, 12, 13, 15
E^1%^_440nm_	199–275 (min 200)	172–190 (min 170)	141–162 (min 120)
E^1%^_257nm_	83–100 (min 70)	74–85 (min 55)	69–79 (min 40)
E^1%^_330nm_	35–49 (20–50)	41–46 (20–50)	39–49 (20–50)

**Table 2:** Prediction of group of storage for the test set samples (*n* = 17) considering the OPLS–DA model performed in Ordoudi *et al.* 2015 study [21] on 98 samples constituting in this work the training set (samples of different geographical origins, harvest year, and stored under different conditions for different periods: “fresh”, 0–4 years of storage and “non-fresh”, 5–14 years of storage). The classification threshold was chosen as 0.6; Y-predicted values equal or greater than 0.6 are highlighted in bold.

Sample	Y Predicted FRESH (0–4 years)	Y Predicted NON-FRESH (5–14 years)
1 *	*0.5*	*0.5*
2 *	*0.5*	*0.5*
3	0.6	0.4
4 *	*0.5*	*0.5*
5	0.4	0.6
6	0.6	0.4
7 *	*0.5*	*0.5*
8	0.7	0.3
9	0.7	0.3
10 *	*0.5*	*0.5*
11	0.3	0.7
12	0.4	0.6
13	0.3	0.7
14 *	*0.5*	*0.5*
15	0.4	0.6
16	0.4	0.6
17	0.6	0.4

* asterisks indicate samples not properly classified, being borderline between the two groups.

**Table 3:** Classification of 17 commercial samples according to the ISO 3632 quality categories [22] soon after the NMR analysis (07/2015).

**Quality Parameters**	**Samples per Commercial Category ^1,2^**		
	I	II	III
	3, 8	1, 4, 6, 17	2, 5, 7, 15 ^2^
E^1%^_440nm_	204–211 (min 200)	169–195 (min 170)	134–162 (min 120)
E^1%^_257nm_	81 (min 70)	70–81 (min 55)	52–69 (min 40)
E^1%^_330nm_	42–49 (20–50)	45–50 (20–50)	33–48 (20–50)

^1^ Samples 9, 10 were no longer available; ^2^ samples 11–14 and 16 were substandard.

**Table 4:** Labeling information for the 17 commercial samples under study.

Sample Number	Labeling Information
1	Purchased from: Hypermarket (Qatar); Origin: Iran; Pack.: September 2013; Exp.: September 2015
2	Purchased from: Open market (Qatar); Origin: Iran; Pack.: October 2013; Exp: October 2015
3	Purchased from: Open market (Qatar); Origin: Iran; Pack.: December 2013; Exp.: December 2015
4	Purchased from: Open market (Qatar); Origin: Iran; Pack.: March 2013; Exp.: March 2015
5	Purchased from: Hypermarket (Qatar); Origin: Iran; Pack.: March 2013; Exp.: March 2015
6	Purchased from: Hypermarket (Qatar); Product of Spain; Pack.: September 2013; Exp.: September 2015
7	Purchased from: Open market (Qatar); Origin: -; Packed in Australia (NSW) Pack.: October 2012; Exp.: October 2014
8	Purchased from: Open market (Qatar); Origin: -; Pack.: -; Exp.: -
9	Purchased from: Super market (Italy); Origin: -; Packed in Italy; Pack.: -; Exp.: 27 January 2017
10	Purchased from: Super market (Italy); Origin: -; Packed in Italy; Pack.: -; Exp.: 2016
11	Purchased from: Open market (Israel); Origin: Spain; Crop: 2011; Exp.: END 2017
12	Purchased from: Open market (Israel); Origin: -; Pack.: -; Exp.: -
13	Purchased from: Herbal shop (KSA); Origin: Iran; Pack. :November 2012; Exp.: November 2015
14	Purchased from: Herbal shop (KSA); Origin: Iran; Pack.: -; Exp.: -
15	Purchased from: Herbal shop (KSA); Origin: -; Pack.: -; Exp.: -
16	Purchased from: Retailer (KSA); Origin: Iran; Prod. Date 4/1434H; Exp. Date 4/1436H
17	Purchased from: Supermarket (Qatar); Origin: Iran; Pack.: November 2012; Exp.: November 2014

## Supplementary Materials

Supplementary materials can be accessed at: http://www.mdpi.com/1420-3049/21/3/286/s1.

## Abbreviations

The following abbreviations are used in this manuscript:

DModX: Distance to the model
DMSO: Dimethylsulfoxide
DRIFTS: Diffuse Reflectance Infrared Fourier Transform Spectroscopy
FT-IR: Fourier Transform Infrared Spectroscopy
g: gravitational force
HPLC-DAD: High performance liquid chromatography-diode array detector
MIR: Mid-Infrared
NIR: Near-infrared
NMR: Nuclear Magnetic Resonance
OPLS-DA: Orthogonal Projection to Latent Structures-Discriminant Analysis
PCA: Principal Component Analysis
PDO: Protected Designation of Origin
PLS-DA: Projection to Latent Structures-Discriminant Analysis
rpm: Revolutions per minute
*trans*-4-GG: *trans*-4-digentiobiosyl ester of crocetin
UV-Vis: Ultraviolet-visible spectroscopy
