# Supplementary material for: On the Traceability of Commercial Saffron Samples Using 1H-NMR and FT-IR Metabolomics
Source: Molecules. 2016 Feb 29;21(3):286. doi: 10.3390/molecules21030286 (PMC6274441; doi:10.3390/molecules21030286)
Supplement: Supplementary file 1 [file molecules-21-00286-s001.pdf]

Supplementary Materials: On the Traceability of Commercial Saffron Samples Using ^{1}H NMR and FT-IR Metabolomics

Roberto Consonni, Stella A. Ordoudi, Laura R. Cagliani, Maria Tsiangali and Maria Z. Tsimidou

Chemometric Analysis of FT-IR Data

FT-IR analysis was performed in the transmission mode (April 2015). The spectral data were thoroughly examined in the regions where qualitative or quantitative differences are expected to occur due to storage effects [20]. These regions are highlighted in the FT-IR profile of "fresh" and "non-fresh" saffron in Figure S1.

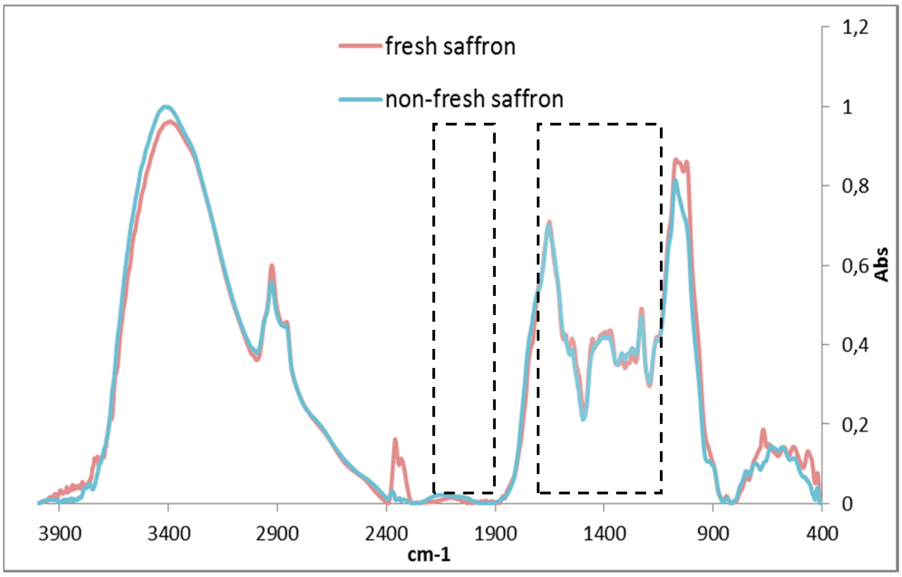

Figure S1. Transmission-FT-IR profiles of authentic, "fresh" and "non-fresh" saffron in the mid-infrared region. Signals in the highlighted regions are attributed to saffron age-related biomarkers.

As expected, the most critical differences were evidenced in the sugar region (1000–1100 cm^{-1}) as well as in the characteristic group region (e.g., C=C, C=N, C=O), 1600–1800 cm^{-1}.

To further exploit these data, the FT-IR database for "fresh" and "non-fresh" saffron presented already by Ordoudi *et al.* [20] was updated with data for new authentic samples, either "fresh" (stored for less than 4 years after processing) and "non-fresh" (stored for 7–12 years).

PCA for the updated data set (n = 53 samples) showed that the first four principal components (PCs) explained the 96% of the total variance (Figure S2). On the basis of score values for the first two PCs, the samples were found to be grouped along the PC2 axis, while PC1 axis does not provide any indication for grouping according to origin, harvest year or conditions of storage. In particular the relative intensities of the bands in the sugar region and more specifically from 1038–1096 cm^{-1} along with that at 1227 cm^{-1} (due to C(O)-O stretching vibrations) positively affected PC2. Most of the "fresh" samples were characterized by positive values higher than 0.08. On the other hand, intensity values in the double bond region (ca. 1632 cm^{-1} as well as in the carbonyl region (1720–1794 cm^{-1}) had highly negative PC2 loadings (≤ −0.08) that accounted for the grouping of the "non-fresh" samples.

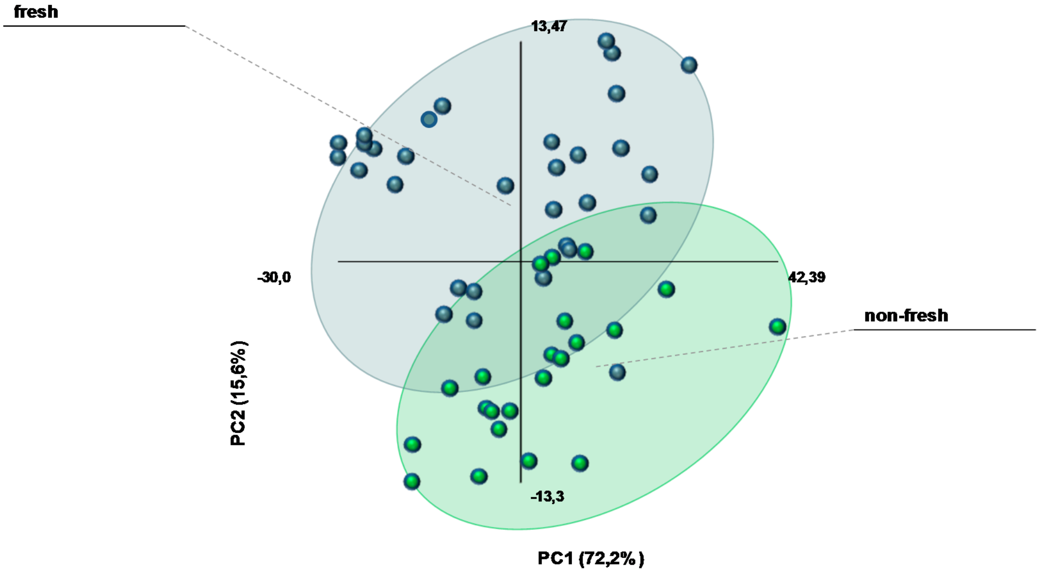

Figure S2. PCA scatterplot of all saffron samples (n=53) of known storage history constituting the database. PC1 = 72.2 %, PC2 = 15.6%, R^2X = 96%, Q^2 = 88%. Blue and green dots represent "fresh" (0-4 years after processing) and "non-fresh" samples (7 - 12 years after processing, respectively).

The plot describing the DModX values of the samples is shown in Figure S3. The sample presenting the greatest DModX value and could be thus considered as possible outlier belonged to the group of the non-fresh ones (12 years old) with sensory characteristics that would easily indicate its deterioration.

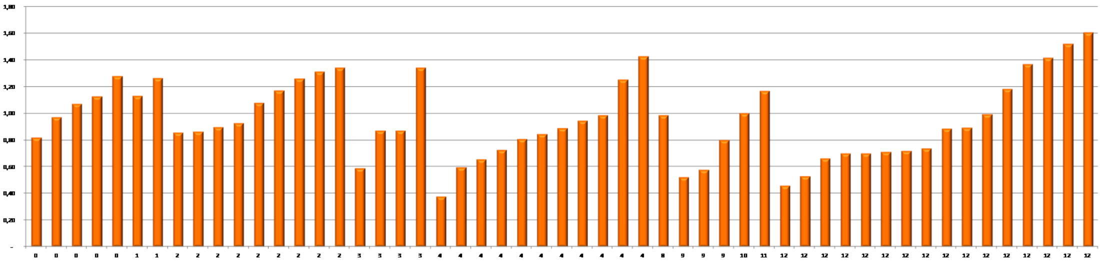

Figure S3. Distance to the model (DModX) values of the training set samples (*n* = 53) obtained after PCA. Values in X-axis represent the age of the sample (years).

The dataset for the 53 samples was then used to perform PLS-DA for improving sample discrimination. However, this treatment resulted in an invalid model with R^2 (*Y*) = 78% and *Q*^{2} = 79%. To check whether this could be due to outliers, we removed from the dataset the sample with the highest DModX value and proceeded with the PCA and PLS-DA of the remaining dataset (*n* = 52). Figure S4a,b shows the corresponding scatterplots.

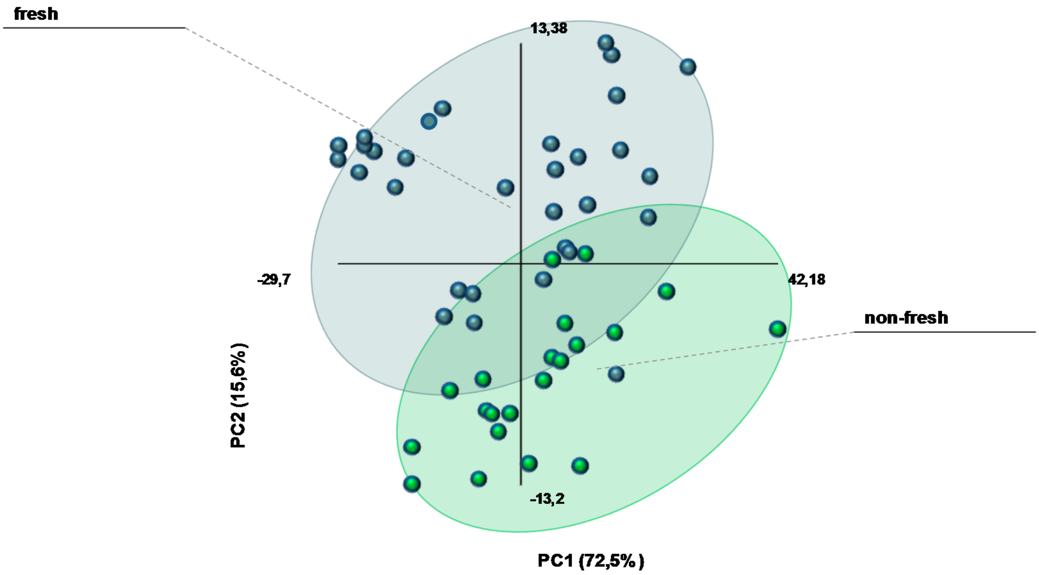

(**a**)

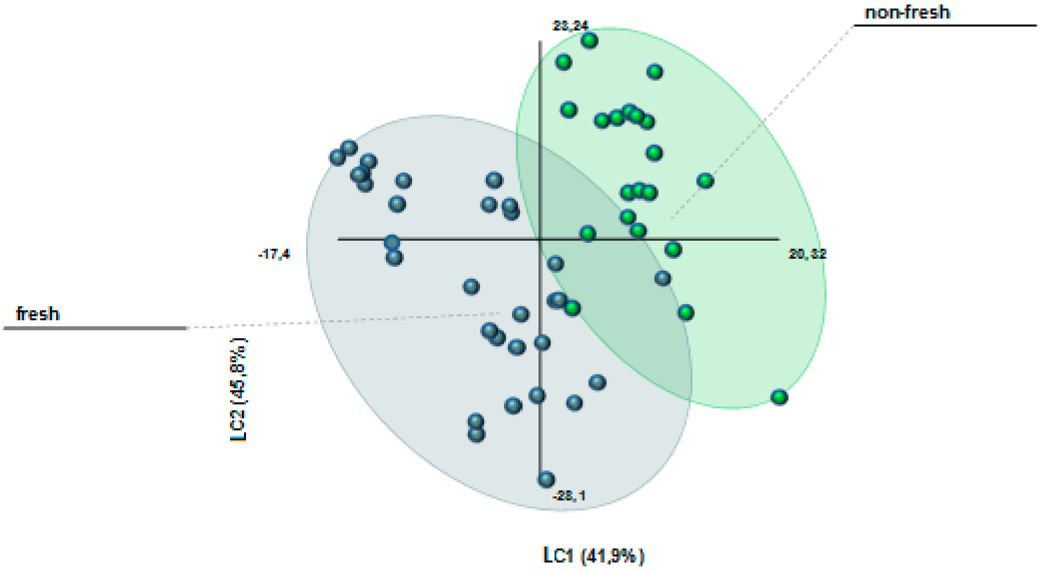

(**b**)

Figure S4. (**a**) PCA scatterplot of the 52 saffron samples constituting the database after the removal of one outlier. PC1 = 72.5 %, PC2 = 15.6%, R^2X = 96%, Q^2 = 89%; (**b**) PLS-DA scatterplot of the training set samples (n = 52) consisting of 31 "fresh" and 21 "non-fresh" ones. R^2 (Y) = 78%, Q^2 = 77%. Blue and green dots represent "fresh" (0–4 years after processing) and "non-fresh" samples (7–12 years after processing).

This model was considered valid after 200 permutations (R^2Y = 78%, Q^2 = 77%) and contained two latent components (LCs) with the first one responsible for the differentiation among samples. In particular, all of the samples belonging to the group of "non-fresh" saffron had highly positive score values for LC1 (t1 >2.0) and most of them had also highly positive values for LC2 (t2 > 6.0). On the other hand, most of the "fresh" samples were distributed to the negative values for LC1 (t1 ≤ 0) while LC2 values ranged mainly within −1 ≥ t2 ≥ −28) (Figure S4b). The variance of band intensities in the regions of 1211–1238, 1009–1107, 824–908 and 962–982 cm^{-1} were found to be most relevant (p1 ≥ −0.09) for the first latent component, as shown in Figure S5. This latter observation confirms previous suggestions that bands in the sugar region are important markers for saffron freshness [20]. Intensity values of specific bands in the sugar region (1067–1074 cm^{-1}) along with those in the region of 1221–1231 cm^{-1} were found to be among the most significant ones (p < 0.05) for the sample differentiation, as indicated by the plot in Figure S6. The variable map shows how these variables contribute to the differentiation between the two groups. The X axis indicates the average ratio

between the two groups while Y axis means–Log(P). The upper-right and left-down corner are the most significant, meanwhile the variables around origin do not contribute to the differentiation.

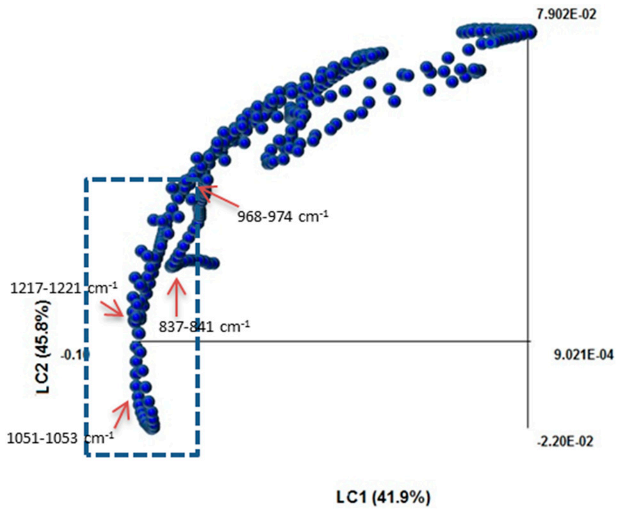

Figure S5. Loading scatterplot of the variables after PLS-DA of the training set samples (n = 52) consisting of 31 "fresh" and 21 "non-fresh" ones. Variables with the highest loading on LC1 are annotated in the plot. The direction of the loading plot corresponds to the same direction in the score plot shown in Figure S4b.

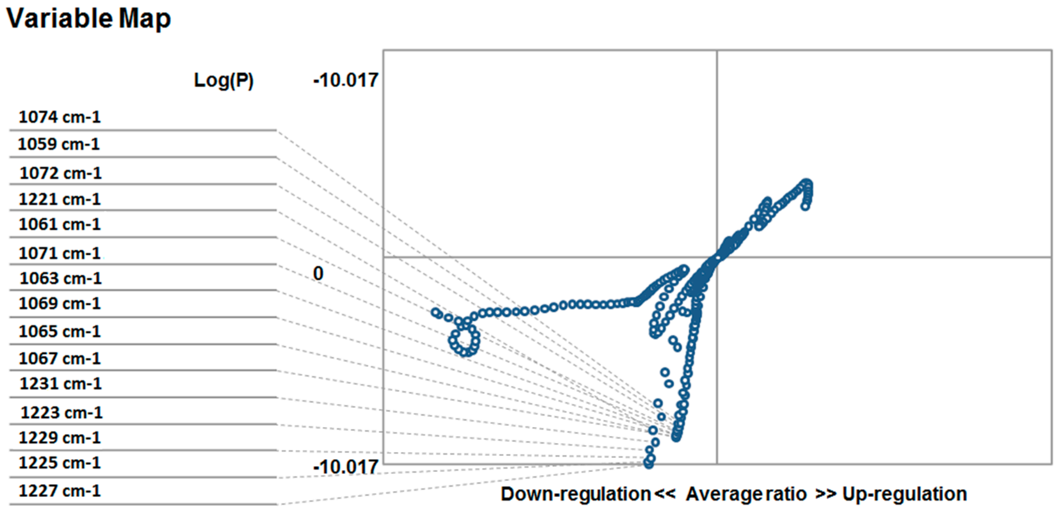

Figure S6. Variables of greatest importance for the discrimination of the samples to "fresh" (n = 31) and "non-fresh" (n = 21). The X axis indicates the average ratio between the two groups while Y axis means–Log(P). The upper-right and left-down corner are the most important for discrimination; meanwhile the variables around origin do not contribute to the differentiation.

A list of the t1 and t2 score values for each of the tested samples is presented in Table S1. Classification was made based on the cut-off values of t1 >2.0 and t2 > 6.0 for "non-fresh" saffron as well as t1 ≤ 0 and t2 ≤ −1 for "fresh" saffron.

Table S1. t1 and t2 score values obtained from PLS-DA of FT-IR data for the commercial saffron samples under study and classification to "fresh" or "non-fresh" saffron.

Sample No	t1	t2	Possible Class Membership [1]
1	2.5	8.5	non-fresh
2	7.8	−7.4	n.a. [2]
3	6.9	−2.9	n.a.
4	1.6	−2.8	n.a.
5	30.8	−5.0	non-fresh
6	0.1	8.8	n.a.
7	4.5	−0.6	non-fresh
8	−3.2	−2.3	fresh
9	−0.2	9.1	n.a.
10	−2.8	12.6	non-fresh
11	5.4	3.2	non-fresh
12	2.0	3.0	non-fresh
13	−0.1	23.0	non-fresh
14	−3.2	15.9	non-fresh
15	6.0	10.3	non-fresh
16	1.7	19.4	non-fresh
17	−1.0	−2.9	fresh

[1] Based on the cut-off values of t1 >2.0 and t2 > 6.0 for "non-fresh" saffron as well as t1 ≤ 0 and t2 ≤ −1 for "fresh" saffron; [2] not clearly assigned.
